# Supplementary material for: Photo- and Thermocatalytic CO2 Methanation: A Comparison of Ni/Al2O3 and Ni–Ce Hydrotalcite-Derived Materials under UV and Visible Light
Source: Materials (Basel). 2023 Aug 29;16(17):5907. doi: 10.3390/ma16175907 (PMC10488339; doi:10.3390/ma16175907)
Supplement: Supplementary file 1 [file materials-16-05907-s001.zip › materials-2517616-supplementary.pdf]

Supplementary material:

Article: Photo- and Thermocatalytic CO_2 Methanation: A Comparison of Ni/Al_2O_3 and Ni-Ce Hydrotalcite-Derived Materials under UV and Visible Light

Materials for catalyst synthesis

- $Ni(NO_3)2{\cdot}6H_2O$ (Sigma-Aldrich)
- $Mg(NO_3)2{\cdot}6H_2O$ (Honeywell)
- $Al(NO_3)_3{\cdot}9H_2O$ Merck (Labkem)
- $Ce(NO_3)_3{\cdot}6H_2O$ (Sigma-Aldrich)
- γ-$Al_2O_3{\cdot}$(Alfa Aesar)
- $Na_2CO_3{\cdot}H_2O$ (Panreac)
- NaOH (Honeywell)
- Ammonia 25 % (as NH_3) (Panreac)

The following figures and tables are added as supplementary information.

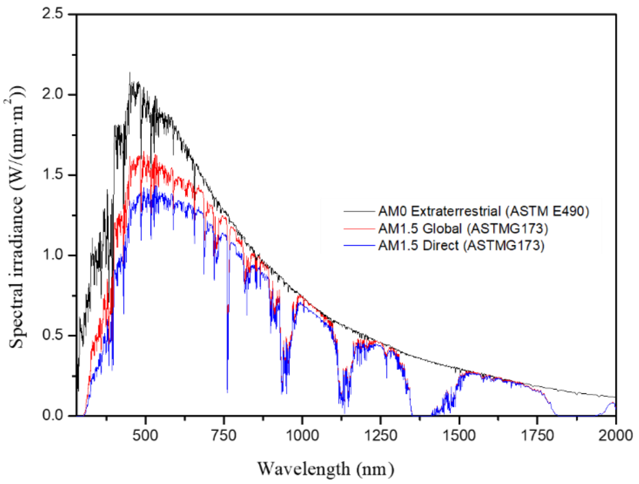

Figure S1. Standard solar spectra for space and terrestrial use.

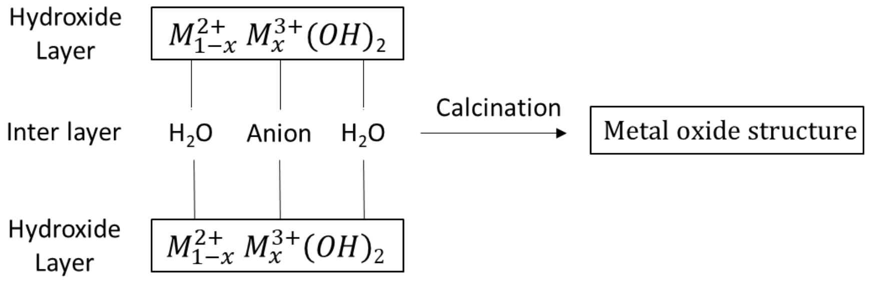

Figure S2. Scheme of the structure of the hydrotalcite before and after calcination.

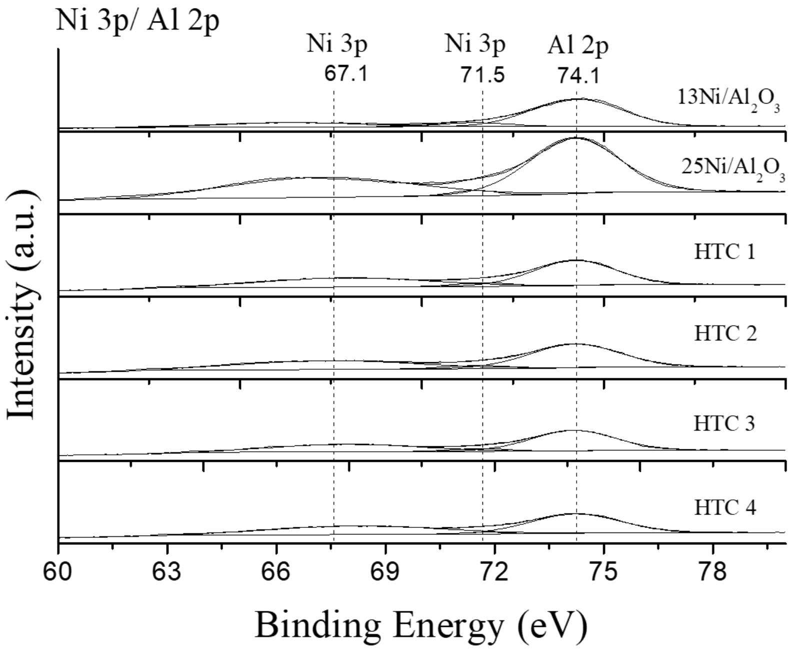

Figure S3. Al 2p-Ni 3p spectra.

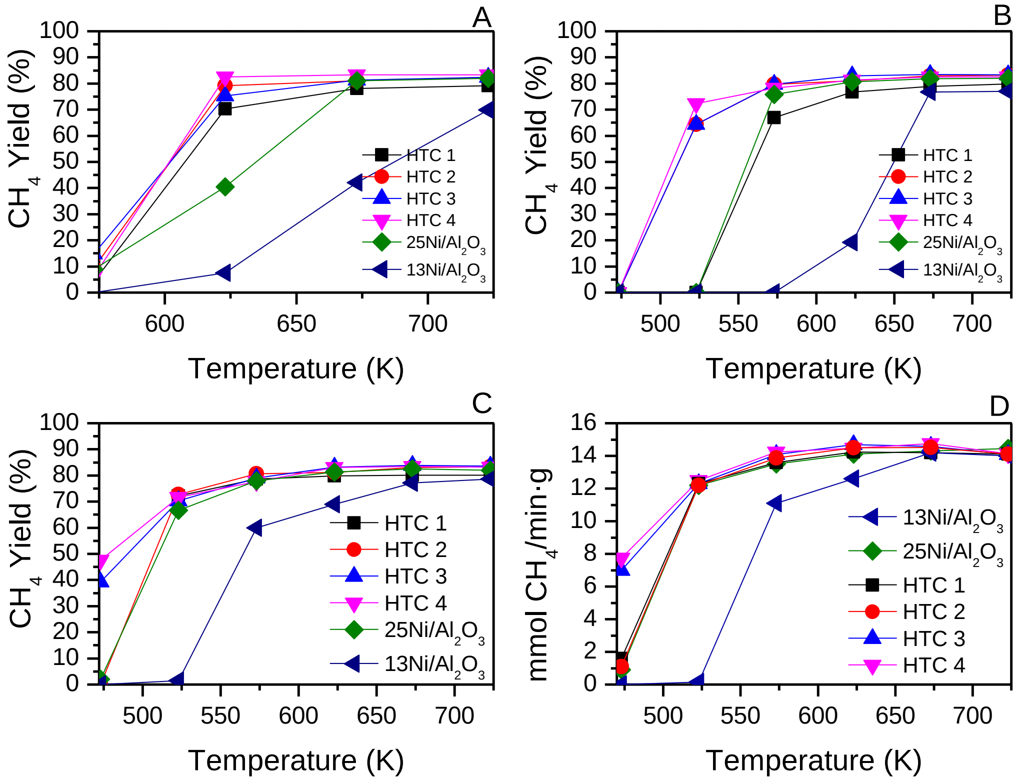

Figure S4. Methane production rate with visible light. Comparison of CH_4 yield enhanced by temperature dark (A), UV light (B), and visible light (C). Methane production rate with visible light (D).

Table S1. Ni $2p_{3/2}$ and Ce $3d_{5/2}$ binding energy of the reduced catalysts.

	Ni^0	Ni^{2+}	Ni^{2+}_{sat}	Ce^{3+} (x_2 and x_4)	Ce^{4+}(x_1 and x_3)
13Ni/Al_2O_3	852.9	857.0	862.3		
25Ni/Al_2O_3	852.2	855.9	861.4		
HTC 1	853.2	856.8	862.9		
HTC 2	852.5	856.4	862.1	880.6–885.5	882.2–888.7
HTC 3	853.0	856.6	861.9	880.5–886.3	882.8–888.1
HTC 4	852.8	856.4	862.0	880.6–885.6	882.7–888.9

Table S2. Surface composition (XPS) and bulk composition (EDS) of the used samples.

	**Atomic composition (XPS)**		**Atomic composition (STEM-EDS)**	
	**Ce/Al**	**Ni/Al**	**Ce/Al**	**Ni/Al**
**25Ni/Al_2O_3**		0.159		0.263
**13Ni/Al_2O_3**		0.069		0.105
**HTC 1**		0.221		0.375
**HTC 2**	0.046	0.263	0.0786	0.375
**HTC 3**	0.075	0.291	0.125	0.366
**HTC 4**	0.101	0.185	0.140	0.345

Characterization of catalysts

Textural properties: N_2 adsorption-desorption isotherms at 77 K were measured for analyzing the specific surface area, pore volume and pore size distribution by an Autosorb 1C-TCD. In order to remove moisture and any adsorbed gases, samples were firstly degassed at 573 K for 2h.

ICP-OES: Metal analysis employed for catalysts was a Perkin Elmer Optima 2000 OV measuring the Ni, Al, Mg and Ce contents. The solid samples were firstly digested by aqua regia (mixture of 3:1 HCl and HNO_3 respectively) at 453 K and then analyzed for identifying the composition and chemical elements according to their characteristic wavelength emission intensity.

H_2-TPR: The reducibility of the catalysts was measured by the temperature programmed reduction employing the Autochem. II Instrument (Micromeritics, USA) Samples were firstly degassed at 383 K for 30 min, cooled down to room temperature and finally, heated up to 1173 K in the case of HTCs and 873 K for Ni/Al_2O_3 carried out over 5 % H_2/Ar flow (30. ml·min^{-1}).

CO_2-TPD: Temperature programmed desorption was performed on the same device as H_2-TPR for surface basicity characterization. The sample was degassed in helium at 573K for 2 h an then cooled at 353 K prior to adsorption of CO_2 at this temperature. After the adsorption of CO_2 (25 ml·min^{-1}) for 1 h the sample was treated with He (40 ml·min^{-1}) for 1h at same temperature in order to remove the physical adsorbed CO_2 from the surface. The desorption curve was recorded at a heating rate of 10 K/min from 353 K to 773 K under He (15 ml·min^{-1}).

XRD: Structural characterization of powder XRD patterns were recorded on a PANalytical Xpert Pro diffractometer using a λCuK_α radiation =0.15418 nm at 40kV and 40mA between 2θ=5º to 80º for identifying the crystalline species.

H_2-chemisorption: Metal dispersion (%) and metallic surface area were determined via H_2 chemisorption method using a Micromeritics Autochem-II. Prior to the analysis, 25 mg of calcined catalyst was pre-reduced according to TPR-H_2 studies for 2 h using 50ml·min^{-1} of 5 vol%

H_2 in Ar. Then H_2 chemisorption analysis was made at room temperature using the same reducing gas.

XPS: X-ray Photoelectron Spectroscopy (XPS) measurements were performed on a SPECS system (Berlin, Germany) equipped with Phoibos 150 1D-DLD analyzer and monochromatic Al Kα(1486.7 eV) radiation source. Spectra were fitted using CasaXPS 2.3.16 software, which models Gauss-Lorentzian contributions, after background subtraction (Shirley). Concentrations were calculated by correcting the values with relative atomic sensitivity factors (Scofield).

UV-Vis DRS: The absorption signal in series of the calcined catalysts were determined by a Shimadzu 3600 UV-vis spectrophotometer collecting data between 200 and 800 nm.

STEM-EDS: All HTCs and Ni/Al_2O_3 catalysts were studied by the Analytical Titan operates in STEM mode at voltages between 60 and 300 kV combined with energy-dispersive X-ray spectroscopy (EDS) with a magnification of 320 K.
